# A prospective, single-centre, randomised study evaluating the clinical, imaging and immunological depth of remission achieved by very early versus delayed Etanercept in patients with Rheumatoid Arthritis (VEDERA)

**DOI:** 10.1186/s12891-016-0915-0

**Published:** 2016-02-05

**Authors:** Raluca B. Dumitru, Sarah Horton, Richard Hodgson, Richard J. Wakefield, Elizabeth M. A. Hensor, Paul Emery, Maya H. Buch

**Affiliations:** Leeds Institute of Rheumatic and Musculoskeletal Medicine, University of Leeds, Leeds, UK; NIHR Leeds Musculoskeletal Biomedical Research Unit, Leeds Teaching Hospitals NHS Trust, Leeds, UK; University of Manchester Centre for Imaging Sciences, Manchester, UK

**Keywords:** Rheumatoid arthritis, Disease modifying anti-rheumatic drug, Etanercept, Treat to target, Remission, Clinical efficacy, MRI synovitis score, Molecular predictors of response

## Abstract

**Background:**

Rheumatoid arthritis (RA) is a chronic inflammatory arthritis, with significant impact on quality of life and functional status. Whilst biologic disease modifying anti-rheumatic drugs (bDMARD) such as tumour necrosis factor-inhibitor (TNFi) agents have revolutionised outcomes in RA, early diagnosis with immediate conventional therapy, titrated in a treat to target approach is also associated with high remission rates. The main aim of the VEDERA study (Very Early versus Delayed Etanercept in Rheumatoid Arthritis) is to assess the depth of remission, sustainability of remission and immunological normalisation induced by very early TNFi with etanercept (ETN) or standard of care +/- delayed ETN.

**Methods/Design:**

VEDERA is a pragmatic, phase IV single-centre open-label randomised superiority trial of 120 patients with early, treatment-naive RA. Patients will be randomised 1:1 to first-line ETN and methotrexate (MTX) or MTX with additional synthetic disease modifying anti-rheumatic drugs (sDMARDs) according to a treat to target (TT) protocol with further step up to ETN and MTX after 24 weeks if remission is not achieved. Participants will have regular disease activity assessments and imaging evaluation including musculoskeletal ultrasound and MRI. The main objective of this study is to assess the proportion of patients with early RA that achieve clinical remission at 48 weeks, following either treatment strategy. In addition, the participants are invited to take part in a cardio-vascular sub-study (Coronary Artery Disease in RA, CADERA), which aims to identify the incidence of cardiovascular abnormalities in early RA.

**Discussion:**

The hypothesis underlining this study is that very early treatment with first-line ETN increases the proportion of patients with rheumatoid arthritis achieving clinical remission, in comparison to conventional therapy.

**Trial registration:**

NCT02433184, 23/04/2015

## Background

Rheumatoid arthritis (RA) is a chronic inflammatory arthritis, characterised by symmetrical, often erosive, inflammatory polyarthritis of the small and medium sized joints, which can lead to significant disability and decreased function [1]. The overall burden of disease however extends beyond the joints to include wide-ranging extra-articular manifestations and comorbidities [2].

The fundamental paradigm in the management of RA is that early, effective suppression of synovitis by early diagnosis and immediate initiation of disease modifying anti rheumatic drug (DMARD) therapy is associated with improved outcomes. Guidelines recommend synthetic DMARD (sDMARD) as a first line therapy with step-up to biological DMARD (bDMARD) treatment in the presence of continued disease activity. Internationally agreed guidelines recommend a treat to target (TT) approach, a concept established for the management of pathologies such as arterial hypertension and diabetes [3]. In RA, TT implies frequent monitoring using a composite disease activity measure such as the disease activity score-28 joint (DAS28) and escalation of therapy according to a pre-defined target of remission or at least low disease activity (LDA) [4, 5]. Tight control of disease activity with intensive treatment, either step-up [6] or parallel [7, 8] in early RA is associated with an improvement in medium and long term outcomes.

The introduction of bDMARDs has provided highly effective therapy with which to achieve effective disease control [9–11]. There are currently five licensed tumour-necrosis factor-inhibitor (TNFi) therapies, the first bDMARDs to be approved for use, of which etanercept (ETN) is a recombinant TNF receptor fused to a human Fc molecule forming a bivalent TNF binding agent. ETN reduces inflammation, improves functional status and quality of life and inhibits radiographic progression [12, 13]. As with other TNFi, ETN is recommended following MTX-inadequate response [14], and in the UK after failure of at least two sDMARDs (of which one should be MTX) [15].

Recent studies including COMET [16], ASPIRE [17] and OPTIMA [18], suggest that TNFi therapy in the early phase of the disease, prior to failure of sDMARDs, offers improved rates of response and inhibition of radiographic progression in comparison to sDMARD. Sub-analysis of the COMET study shows that patients receiving very early treatment with ETN and methotrexate (within the first 4 months of disease) achieve greater rates of remission than those with longer disease [19]. Furthermore, studies suggest that when commenced early, TNFi may offer a greater depth of remission that is more likely to be sustained after drug withdrawal [20]. In recent years, increasing consideration has been given to the ‘window of opportunity’ concept: a phase in early disease in which it may be possible to alter the pathogenic course of the disease [21]. However, not all studies demonstrate the superiority of TNFi when compared to TT regimen in early disease; in a group of DMARD-naive early RA patients, no statistically significant differences were found between patients receiving MTX and intravenous steroids, followed by TT regimen and combination therapy with MTX and infliximab [22]. There is thus, continued debate on how best to apply TNFi therapies in order to obtain the maximal benefits to the patient whilst considering socio- and health-economic costs. In addition, increasing research is aimed at identifying predictors of response. This includes use of TNFi within a TT strategy [17, 18], and for remission [23].

An additional challenge is the assessment of therapeutic response. Whilst clinical scores such as DAS28 form the basis of evaluation, it is accepted that these may be insensitive [24]; imaging modalities such as power Doppler (PD) ultrasound (US) and dynamic magnetic resonance imaging (MRI) enable more accurate assessment of inflammation as well as providing insights into the pathobiology of disease [25].

## Methods/Design

### Aims and objectives

The main aim of the VEDERA study (Very Early versus Delayed ETN in Rheumatoid Arthritis) is to evaluate the depth of remission, sustainability of remission and immunological normalisation induced by very early TNFi or the current standard of care (with or without delayed TNFi if required), in patients with early, treatment-naive RA.

#### Research hypothesis

Very early treatment with first-line ETN increases the proportion of patients with rheumatoid arthritis achieving clinical remission, in comparison to conventional therapy.

#### Primary objective

The primary objective of this study is to assess the proportion of patients with early, treatment-naive RA that achieve clinical remission at 48 weeks following first-line ETN and MTX (‘very early ETN’) or MTX with additional sDMARDs and step up to ETN and MTX at 24 weeks, as required according to a TT protocol aiming for remission (‘delayed ETN’).

#### Secondary objectives

- To assess the change in MRI synovitis between baseline and 48 weeks following very early ETN or MTX-TT regimen +/- delayed ETN.
- To evaluate other clinical efficacy endpoints at weeks 12, 24, 48 and 96 including:
  - ✓ Disease activity score-44 joint (DAS44) remission (DAS44 < 1.6), simplified disease activity index (SDAI), clinical disease activity index (CDAI), European League Against Rheumatism (EULAR) response criteria, American College of Rheumatology (ACR) response criteria.
  - ✓ Cumulative steroid dose.
- To evaluate patient-reported outcomes at weeks 12, 24, 48 and 96 including:
  - ✓ Physical function, assessed by the Health Assessment Questionnaire (HAQ), including normalisation of HAQ.
  - ✓ Quality of life scores assessed by RA Quality of Life (RA-QoL) and Euro QoL Five Dimensions (EQ-5D) questionnaires.
  - ✓ Work instability, assessed by RA Work Instability Score (RA-WIS) questionnaire.
- To evaluate imaging predictors of remission and sustained remission using:
  - ✓ High-resolution ultrasound (HRUS) at weeks 0, 12, 24 and 48 measuring grey scale (GS), PD and presence or absence of erosions.
  - ✓ Magnetic Resonance Imaging (MRI) at weeks 0, 12, 24 and 96 measuring synovitis and erosion scores.
- To evaluate other radiographic outcomes including:
  - ✓ Joint damage assessed by modified Sharp score of X-rays of hands and feet at weeks 48 and 96.

#### Exploratory objectives

- To investigate immunological abnormalities and cellular/molecular predictors of response to the two therapeutic strategies using:
  - ✓ Blood, sampled at baseline and weeks 12, 24, 36, 48 and 96.
  - ✓ Synovial tissue (optional), biopsied from a target joint at baseline and 24 weeks; at 48 weeks for treatment arm 2 who have switched to ETN and at time of flare.

### Study design

The VEDERA study is a phase IV single-centre open-label randomised trial of 120 patients with early, treatment-naive RA. Patients will be randomised 1:1 to first-line ETN and MTX or MTX with additional sDMARDs and step up to ETN and MTX after 24 weeks as required according to a TT protocol aiming for remission.

The randomised trial duration is 48 weeks after which ETN will be stopped and patients will continue for a 48-week observational period during which DMARDs will be used as per standard care, in accordance with national guidelines.

### Patient eligibility

The target population are males and females, aged between 18 and 80 years, fulfilling the 2010 American College of Rheumatology/European League against Rheumatism (ACR/EULAR) RA classification criteria, who have not yet received DMARD therapy, with a maximum symptom duration of 12 months. Other criteria for inclusion are: patients with active RA at baseline (clinical evidence of synovitis and DAS28-ESR > 3.2) and positive anti-citrullinated peptide antibody (ACPA) or rheumatoid factor (RF) status. In the absence of RF and ACPA antibodies, patients are eligible for the study if they have evidence of power Doppler, a good indicator of active inflammatory pathology and predictor of structural damage. Power Doppler grade ≥1 in at least 1 joint on hand ultrasound is required (Table 1).

**Table 1:** Eligibility criteria for randomisation into VEDERA trial

Inclusion Criteria
• Male and female patients aged between 18 and 80 years.
• Diagnosis of rheumatoid arthritis (new 2010 ACR/EULAR RA classification criteria).
• Symptom onset within the preceding 12 months.
• Patients with active RA at baseline: clinical evidence of synovitis (or imaging-evidence of synovitis in cases of uncertainty/subclinical disease) in hand and/or wrist joints evaluable by ultrasound and MRI, and DAS28-ESR > 3.2.
• Seropositivity for anti-citrullinated peptide antibody (ACPA) and/or rheumatoid factor. If ACPA and rheumatoid factor are both negative, presence of power Doppler (grade 1 or higher) in at least 1 joint on hand ultrasound is required.
• DMARD-naive (with the exception of previous exposure to hydroxycholorquine for an indication other than RA).
• All male and female subjects biologically capable of having children must agree to use a reliable method of contraception for the duration of the study and 24 weeks after the end of the study period. Acceptable methods of contraception are surgical sterilisation, oral, implantable or injectable hormonal methods, intrauterine devices or barrier contraceptives.
Exclusion Criteria
• Previous treatment with DMARDs for the management of RA.
• Intramuscular or intra-articular (of non-target joint) corticosteroid within 28 days of the screening visit; intra-articular steroid of the chosen target joint within 12 weeks of screening.
• Oral steroid of greater than 10mg prednisolone daily, or change in oral steroid dose within 28 days of study drug initiation at the baseline visit.
• Use (including use as required) of more than one NSAID, change in NSAID or change in dose of NSAID within 28 days of the baseline visit.
• Contraindications to MRI (e.g. pacemaker) or unable or unwilling to attend for all imaging assessments. In patients with previous penetrating trauma to the eye, or patients at high risk of previous metal foreign body injury to the eye (e.g. welding), skull x-ray will be performed; these patients may be included in the absence of residual metal fragments on x-ray.
• Pregnancy or breastfeeding.
• Other contraindications to TNFi as determined by local prescribing guidelines and physician discretion, including: active infection, open leg ulcers, previously infected prosthetic joint (unless completely removed), septic arthritis in last year, HIV, Hepatitis B or Hepatitis C carriers, previous malignancy within 10 years (except basal cell carcinoma), severe heart failure (New York Heart Association grade 3 or more), any history of demyelinating disease, uncontrolled diabetes, pulmonary fibrosis, bronchiectasis, previous PUVA therapy (of >1000 Joules), history of TB or evidence of latent TB on chest x-ray/TB testing (in the latter event, a patient may be included if treated with isoniazid and pyridoxine one month before starting the study and for a further 6 months whilst on study treatments).

### Recruitment

Participants will be recruited from the rheumatology clinics based within Leeds Teaching Hospitals NHS Trust. The recruitment period is expected to last up to 36 months. All patients in the VEDERA study will be invited to participate in CADERA, the cardiovascular sub-study.

### Screening

Following written informed consent and prior to any trial related procedures, participants will be registered and undergo a screening assessment that should occur no more than four weeks prior to the baseline assessments, to determine eligibility for the study.

### Randomisation

**Fig. 1:**
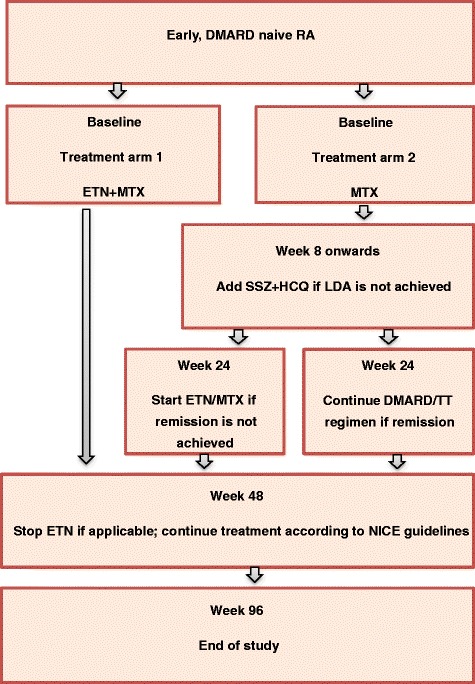
Schematic of the trial design. DMARD disease modifying antirheumatic drugs, ETN etanercept, HCQ hydroxychloroquine, LDA low disease activity, MTX methotrexate, NICE National Institute of Clinical Excellence, RA rheumatoid arthritis, SSZ sulfasalazine, TT treat to target

Once eligibility is confirmed and all relevant procedures have been completed, participants will be block randomised 1:1 to one of the following two treatment groups (Fig. 1):

- Treatment Arm 1 or ‘very early ETN’ will receive ETN and MTX combination therapy administered for a total duration of 48 weeks.
- Treatment Arm 2 or ‘delayed ETN’ will receive initial MTX monotherapy with adoption of a TT protocol (standard care involving monthly DAS28-ESR assessment with escalation to combination sDMARD therapy if not achieving LDA at, or after, 8 weeks) and step-up to ETN and MTX at 24 weeks if failing to achieve clinical remission.

### Trial intervention

#### Treatment arm one

For treatment arm one (Table 2), ETN will be administered subcutaneously up to week 48 (except in the case of intolerance or subject withdrawal) at a dose of 50 mg weekly. MTX will be administered orally at a starting dose of 15 mg weekly, increasing to 20mg and 25mg weekly at weeks 4 and 8 respectively. Patients that are clear non-responders or intolerant to ETN and/or MTX will be managed according to physician discretion (withdrawing these study treatments if appropriate).

**Table 2:** Treatment arm scheme

Arm 1	Dosage form	Dosage regimen	Duration
*ETN*	Subcutaneous	50mg weekly	Up to week 48 unless non-response or intolerance.
*MTX*	2.5/10mg tablets (or s.c. if oral not tolerated)	Weeks 0-4: 15 mg weekly Week 4: 20 mg weekly Week 8 onwards: 25 mg weekly	For duration of study (unless intolerance: aim for maximum tolerated dose)
*Folic Acid*	5mg tablets	5mg, six days per week (not day of MTX)	For duration of study (if receiving MTX)
Arm 2			
*MTX*	2.5/10mg tablets (or s.c. if oral not tolerated)	Weeks 0-2: 15 mg weekly Week 2 onwards: 25 mg weekly	For duration of study (unless intolerance, aim for maximum tolerated dose)
*Folic Acid*	5mg tablets	5mg six days per week (not day of MTX)	For duration of study (if receiving MTX)
*SSZ*	500 mg tablets	1 g twice a day	At/after week 8 if not achieving low disease activity (DAS28ESR > 3.2). Discontinuation if ETN is started at week 24.
*HCQ*	200mg tablets	200mg tablets	At/after week 8 if not achieving low disease activity (DAS28ESR > 3.2). Discontinuation if ETN is started at week 24.
*ETN*	Subcutaneous	50mg weekly	At week 24 to week 48 if not achieving remission (DAS28ESR ≥ 2.6) Thereafter continuation will be determined by physician judgement/according to national guidelines.

*ETN* Etanercept, *MTX* Methotrexate, *SSZ* Sulfasalazine, *HCQ* Hydroxychloroquine

#### Treatment arm two

In treatment arm two (Table 2), MTX will be administered orally at a starting dose of 15 mg and will be increased to 25mg weekly at 2 weeks. Subcutaneous MTX may be administered if intolerance to oral MTX is observed. If at weeks 8, 12, 16 or 20, the subject fails to achieve LDA (defined as DAS28-ESR ≤ 3.2), sulfasalazine (SSZ) and hydroxychloroquine (HCQ) will be added to MTX. SSZ will be administered orally at a dose of 1g twice daily (or at the maximum tolerated dose). HCQ will be administered at a dose of 200mg daily. At 24 weeks, if a subject fails to achieve clinical remission, ETN will be added to MTX, and SSZ and HCQ will be discontinued (if applicable).

#### Treatment arms 1 and 2

Oral folic acid will be administered at a dose of 5mg daily (except on the day of MTX) to subjects in both treatment arms.

ETN will be discontinued in both arms at the primary endpoint (48 weeks), with the exception of those patients who are eligible to continue according to local prescribing guidelines (NICE guidelines) [15].

A single non-steroidal anti-inflammatory drug (NSAID) is permitted providing the dose has been unchanged for at least 28 days prior to study drug initiation at baseline. The NSAID or NSAID dose may be changed during the time course of the study if indicated.

Oral prednisolone is permitted at doses up to and including 10mg prednisolone daily if the dose has been stable for at least 28 days prior to study drug initiation at baseline. The steroid dose may be reduced throughout the study.

Intramuscular steroid may be administered as per the study treatment protocol for each arm with all patients receiving 120mg intramuscular methylprednisolone (unless contraindicated or not tolerated) at baseline and at the following time-points according to treatment arm and disease activity:

- Week 12: both treatment arms, if DAS28-ESR > 3.2
- Week 24: treatment arm 1, if DAS28-ESR ≥ 2.6 (treatment arm 2 switch to ETN+/-MTX if DAS28-ESR ≥ 2.6)
- Week 36:
  - ✓ Treatment arm 1, if DAS28-ESR≥2.6
  - ✓ Treatment arm 2, sDMARDs (MTX +/-SSZ +/-HCQ), if DAS28-ESR≥2.6
  - ✓ Treatment arm 2, switched to ETN+/-MTX, if DAS28-ESR>3.2
- Up to the primary endpoint (week 48), for a patient with active disease who is judged by the physician to be in need of rescue therapy, and for whom it is considered to be unethical to wait until the 12, 24 and 36 week time points above, 120mg methylprednisolone may be administered.

At and/or after the primary endpoint (week 48) intramuscular or intra-articular steroids are permitted according to physician judgment, unless a clinical or imaging assessment is scheduled within the following 6 weeks.

Alternative DMARDs, other than study treatments, are permitted if clinically indicated in a subject judged by the physician to be a non-responder (primary or secondary non-responder) or intolerant to ETN.

#### Prohibited medications

Steroids are prohibited prior to week 48, with the exception of:

- Patients receiving ≤10mg prednisolone daily with a stable dose for at least 28 days prior to study drug initiation at baseline.
- Patients receiving prednisolone for an indication other than arthritis, for example asthma or chronic obstructive pulmonary disease (up to a maximum total oral dose of 250mg, and a maximum duration of 14 days).
- Intramuscular steroid as per the study treatment protocol for each arm.
- 120mg methylprednisolone administered as rescue therapy (see above).

In the unavoidable instance of a subject receiving corticosteroid within a significant time window prior to a clinical +/- imaging joint assessment the assessment will not be performed. A significant time window is defined as follows: intramuscular corticosteroid within 4 weeks or oral prednisolone within 7 days of the assessment, except patients on stable prednisolone ≤10mg.

At or after week 48, intramuscular or intra-articular steroids are prohibited within 6 weeks of a scheduled clinical or imaging assessment.

Other prohibited medications are any alkylating agents (e.g. cyclophosphamide, chlorambucil), any experimental drugs and immunisations with live vaccines.

### Study schedule

- Screening visit. All patients will undergo screening within the 4 weeks prior to the baseline visit (see section ‘Screening’ above).
- Baseline visit (week 0). This visit will be performed to confirm eligibility for the study and for randomisation and initiation of study treatment.
- Assessment and treatment visits. These visits will be performed at weeks 4, 12 and every 12 weeks thereafter, up to week 96, for both treatment arms.
- Additional visits (for treatment arm 2). The patients in treatment arm 2 will also be monitored at week 8, 16 and 20 for safety and efficacy within a TT protocol. In patients switching to ETN and MTX at week 24 a follow up safety visit will be arranged 4 weeks following ETN commencement (i.e. week 28).

### Methods of assessment

#### Clinical efficacy

Clinical efficacy and remission will be assessed by the following measures, with joint examinations performed by the same blinded assessor.

*DAS44 and DAS28*, based on the evaluation of four variables: tender joint assessment (Ritchie Articular Index, RAI, and 28 joint count respectively), number of swollen joints (out of 44 and 28, respectively), erythrocyte sedimentation rate (ESR; mm/h) and patient’s global assessment of arthritis, as assessed by a 100 mm visual analogue scale (VAS). These will be performed at each visit for both treatment arms. The scores will be calculated using the following formulae: DAS28 = 0.56*sqrt (tender28) + 0.28*sqrt(swollen28) + 0.70*ln(ESR) + 0.014*VAS; DAS 44 = 0.54*sqrt(RAI) + 0.065*(swollen 44) + 0.33*ln(ESR) + 0.0072*VAS. Remission thresholds for DAS44 and DAS28 are <1.6 and <2.6 respectively [26].

*CDAI* is the sum of four variables: number of tender and swollen joints (out of 28), the investigator’s global assessment of disease activity and the patient’s global assessment of arthritis assessed by VAS (cm). The threshold for CDAI remission is <2.8 [27].

*SDAI* is the CDAI plus CRP (mg/dL). The threshold for SDAI remission is <3.3 [28].

*EULAR response criteria* classify patients as good, moderate or non-responders based on a combination of the actual DAS and change from previous DAS [29].

*ACR response* measures 20 % (ACR20), 50 % (ACR50) or 70 % (ACR70) improvement in tender and swollen joint counts and in at least three of the following parameters: patient global assessment, physician global assessment, pain, disability and acute phase reactant (ESR or CRP) [30].

*The physician global assessment* of rheumatoid disease activity should be completed before the patient’s global assessment is received. The investigator will mark their assessment on a 100 mm visual analogue scale (VAS): the left end corresponds to none (0) and the right end to extremely active (100).

#### Patient-reported outcomes

*Patient general health assessment and pain visual analogue scales (VAS):* the patient is instructed to mark their ‘general state of health’ and ‘level of pain’ on two 100mm scales. The left end (0) corresponds to ‘very well’ and ‘no pain’ respectively, and the right end (100) to ‘extremely poor’ and ‘worst possible pain’ (100).

*The HAQ* assesses a patient’s level of functional ability. There are 20 questions in 8 categories of functioning that represent different activities - dressing, rising, eating, walking, hygiene, reach, grip and usual activities. For each item there is a 4-level difficulty scale scored from 0-3, representing no difficulty (0), some (1) or much (2) difficulty, and unable to do (3). The highest component score in each category determines the category score, unless the patient uses aids or devices for, or receives assistance with activities in that category, in which case the relevant category score is increased to 2 if the maximum score was previously <2. The 8 category scores are averaged into an overall score from zero to 3 [31].

*The RAQoL* consists of 30 statements derived directly from relevant patients, using, as far as possible, their own words. Respondents are required to indicate whether or not each of the statements applies to them; each affirmed statement contributes a score of 1 to the total score. Scores can range from 0 to 30 with a [32].

*The EQ5D* is a generic measure, which provides a single index value [33]. The scale includes a descriptive system, comprising 5 questions relating to different aspects of health each with 3 possible responses (‘no problems’, ‘some problems’ and ‘extreme problems’), and a visual analogue scale capturing the patient’s self-rated health where the endpoints are labelled ‘best imaginable health state’ and ‘worst imaginable health state’.

*The RA-WIS* [34] consists of 23 statements about the impact of the disease on working. Respondents are required to indicate whether or not each of the statements applies to them; each affirmed statement contributes with a score of 1 to the total score. Scores can range from 0 to 23; a score less than 10 indicate low work instability; scores in the range of 10-17 indicate moderate work instability; scores above 17 indicate high risk of work instability.

*Duration of early morning stiffness*; the patient is asked to estimate the time that elapsed between awakening and the time he/she is as flexible as he/she will be during a day involving typical activities. Duration in minutes is recorded up to a maximum of 720 min (12 h).

#### Imaging assessments

*MRI (high field gadolinium enhanced)* of the dominant hand and wrist (or alternative hand in the instance of greater clinical evidence of synovitis at baseline) will be performed at baseline, weeks 12, 24, 48 and 96. The dominant hand will be scanned unless the alternative hand has clinical evidence of greater synovitis at baseline. This study will use MRI to assess erosions, synovitis and bone marrow oedema using the accepted standard of RA magnetic resonance imaging score (RAMRIS), as well as direct synovial volume measurement and sensitive dynamic contrast enhanced magnetic resonance imaging (DCE-MRI) for quantification of synovitis and bone marrow oedema. Failure of MRI acquisition after baseline will not constitute a protocol violation and will not necessitate withdrawal from the study.

RAMRIS scoring system: scoring of erosions, synovitis and bone marrow oedema, using the validated outcome measures in RA clinical trials (OMERACT) system, will be performed by 2 independent, experienced scorers. The RAMRIS scoring system is a standardised, reliable, validated scoring system for synovitis, erosion and oedema. It has been successfully used to demonstrate response to treatment, including bDMARD [35]. Direct volume measurement of synovitis by manual segmentation will be performed. This will allow quantification of low volumes of synovitis not differentiated by RAMRIS scoring.

This will be followed by dynamic contrast enhanced (DCE)-MRI. Early enhancement rates measured from DCE-MRI depend strongly on synovial vascularity and capillary permeability and are therefore potentially better markers of inflammatory activity than simple volume measurements. DCE-MRI measurement of bone marrow oedema will also be performed.

A blinded assessor will perform *HRUS (Philips HDI 5000)* of the same hand and wrist as chosen for MRI at baseline, weeks 12, 24 and 48. If the target joint for synovial biopsy is at another site, the target joint will also be scanned. Images will be assessed for synovitis, using semi-quantitative scores of GS and PD and for presence or absence of erosions.

Plain radiographs of bilateral hands (carpal, MCP and PIP joints) will be performed at baseline, 12 and 24 months after the start of study medication to assess structural damage. Radiographs will be scored as per the modified Genant-modified Sharp scoring system [36].

#### Exploratory biosample based research

Blood samples will be collected at baseline and weeks 12, 24, 36, 48, 96, at time of early discontinuation (if indicated) and at time of flare (if indicated). This will include collection of serum, plasma and heparinised blood (for flow cytometry and functional studies).

Synovial biopsy (optional) will be taken from a target joint: an active joint (identified at baseline by presence of PD activity on ultrasound) or alternatively, if necessary, a clinically uninvolved, accessible joint may be chosen. The optional biopsy will be acquired via arthroscopy or under ultrasound guidance at baseline and week 24; at week 48 in patients randomised to treatment arm 2 receiving delayed ETN from week 24, and at time of flare in the case of loss of response in an initial responder. Failure of biopsy acquisition at any time point will not constitute a protocol violation and will not necessitate withdrawal from the study.

The sample collection will be used for wide-ranging studies including cellular phenotyping, soluble biomarker evaluation and gene expression studies.

A detailed study schedule is provided in Table 3.

**Table 3:** Study schedule

				Arm 2^a^		Arm 2^a^	Arm 2^a^								
Study Week	Weeks -4 to 0	Week 0	Week 4	Week 8	Week 12	Week 16	Week 20	Week 24	Week 36	Week 48	Week 60	Week 72	Week 84	Week 96	Early discontinuation
Study Phase	Screen	Baseline	Safety visit							1^0^ Endpoint				End of Study	
Visit No.	1	2	3	4	5	6	7	8	9	10	11	12	13	14	
Informed Consent (patient information will be provided > 24 hrs prior to screen)	X														
Inclusion/exclusion criteria	X	X													
Randomisation		X													
Demographics, RA Hx, past medical Hx, Family Hx, CV risk factors^b^	X														
Physical examination^c^	X				X										X
Vital signs^d^	X	X	X		X			X	X	X	X	X	X	X	X
Pregnancy test^e^	X	X													
ESR, CRP and HS-CRP	X	X	X (Arm 2 only)	X	X	X	X	X	X	X	X	X	X	X	X
Blood chemistry, haematology	X	X	X	X	X	X	X	X	X	X	X	X	X	X	X
Urinalysis	X	X	X		X			X		X		X		X	X
Serology: RF, ACPA and ANA	X													X	X
ECG	X														
Chest x-ray/TB test^f^	X														
Hepatitis (B and C) serology	X														
VAS assessments: patient general health, patient global, patient pain	X	X	X (Arm 2 only)	X	X	X	X	X	X	X	X	X	X	X	X
Physician VAS global disease activity	X	X			X			X		X		X		X	X
DAS_28/44_ joint assessment^g^	X	X	X (Arm 2 only)	X	X	X	X	X	X	X	X	X	X	X	X
Early morning stiffness, HAQ, RA QoL, EQ-5D, RA-WIS		X			X			X		X		X		X	X

a. Visits at weeks 8, 16 and 20 are only applicable to patients in treatment arm 2. b. Cardiovascular risk factors include: smoking habit (pack years), alcohol intake (units/week), amount of exercise taken, dietary intake (days/week ≥ 5 portions fruit or vegetables consumed), and family history of premature cardiovascular disease age <55 years. c. Physical examination Includes height and body weight at screening. d. Blood pressure after a 5-min rest, pulse rate and body temperature e. Urinary pregnancy test for women of child bearing potential only f. Chest x-ray, if not already performed within 24 weeks prior to the study. TB testing includes TB Gold Quantiferon +/- T spot test +/- Mantoux test. g. Whenever possible, joint assessments should be performed by the same blinded assessor throughout the time course of the study to reduce potential investigator bias

### Follow-up

At study completion (96 weeks), patients will be invited to take part in the Inflammatory Arthritis Continuum (IACON) study. This is an in-house, prospective, longitudinal, observational study of patients with inflammatory arthritis, with ethical approval to obtain blood samples and clinical and imaging data in consenting patients. Inclusion of all patients with inflammatory arthritis in IACON will allow recording of longer-term outcomes such as sustained response and drug-free remission permitting future observational work.

### Statistical considerations

#### Sample size

Clinical remission (DAS28 remission) forms the basis of the primary outcome. The COMET study, reported remission rates in patients with very early RA (less than 4 months since diagnosis) treated with both ETN and MTX or MTX monotherapy of 70 % versus 35 % respectively. In patients with disease duration of less than 2 years but greater than 4 months, treatment with ETN and MTX was associated with 48 % remission rate. In the COMET study patients in the very early RA group were recruited within 4 months of RA diagnosis, using ACR 1987 criteria. In contrast, VEDERA will recruit patients within 12 months of symptom onset. Whilst all patients will meet RA criteria at the point of recruitment, ACR/EULAR 2010 criteria are being used, which identify patients at an earlier stage of the disease course than the 1987 criteria, and patients can potentially be recruited concurrently with diagnosis. It is likely that patients recruited to VEDERA will be at a similar stage in their disease course to the patients in the very early RA group in COMET, potentially an even earlier stage.

To account for the possibility that the interval between the treatment groups may not be as large as that seen in COMET a 30 % difference has been used as a basis for the sample size calculation. Assuming therefore that patients in treatment arm 1 are likely to achieve up to 70 % remission, and treatment arm 2 approximately 40 % remission; for 80 % power at the 5 % significance level and allowing for a 10 % dropout rate, at least 49 patients per arm will be required. Increasing this to 60 per arm would allow for a subgroup analysis of treatment arm 2 patients that proceed to require delayed ETN and MTX to be compared with treatment arm 1 and indeed those in arm 2 that stay on conventional therapy. This is an exploratory analysis; we estimate that 50 % of treatment arm 2 patients will require delayed ETN and MTX. With roughly 30 patients in each subgroup of treatment arm 2 we will obtain sufficiently accurate estimates of remission rates.

#### Analysis populations

The full analysis set will comprise all patients, allocated to treatment groups as originally randomised, irrespective of the treatment received and any subsequent deviations from the study protocol. The per protocol set will comprise patients with primary endpoint data available, whose treatment complies with the study protocol and for whom no other major protocol violations are identified. Protocol violations will be identified during a data review by the study management team, blind to allocation, and prior to locking the study database.

#### Analyses

Descriptive summary statistics will be provided for all variables at baseline and at each visit at which the variable is assessed. For continuous variables the following information will be provided: number of patients (N), mean, standard deviation, minimum, median, maximum and 25 % and 75 % quartiles. For categorical data frequency (absolute and relative) distributions will be provided. Differences between groups will be summarized according to data type and 95 % confidence intervals around these estimates will be constructed.

All inferential analyses will be conducted using two-tailed tests at the 5 % level of significance. The Holm correction (modified Bonferroni) will be employed on a family-wise basis to control for multiple comparisons of secondary outcomes.

Complete details of statistical analyses and methods, including data handling conventions, will be contained in a separate statistical analysis plan which will be finalized before the database is locked.

##### Primary efficacy analysis

The primary efficacy analysis will be conducted in the full analysis set. A Pearson’s Chi-squared test will be used to compare the proportions of subjects achieving clinical remission between the two treatment arms. The 95 % confidence interval for the odds of achieving clinical remission at week 48 will also be constructed. Analyses will be repeated in the per protocol set.

##### Secondary efficacy analyses

Secondary efficacy analyses will be conducted in the full analysis set. Proportions of patients achieving remission or other binary responses will be compared between groups using Pearson’s Chi-squared tests. Changes in Gaussian-distributed continuous interval variables over time will be compared between groups using mixed between-within subjects ANOVA; if the data do not meet the assumptions of the test, approaches such as rank transformation will be used. Time to event analyses will be conducted using log-rank tests. Analyses will be repeated in the per protocol set.

##### Other planned analyses

Immunological, gene expression and histological parameters will be compared between the two treatment arms using the same approaches listed for the secondary analyses with appropriate bioinformatics as indicated; subgroup analyses will repeat the between-arm comparison, split by remission status (achieved/not achieved).

Clinical, imaging, synovial and immunological factors associated with the odds of early and sustained remission will be investigated using univariable and multivariable binary logistic regression models.

RAMRIS score is considered a secondary efficacy outcome and will be analysed accordingly. For the remaining exploratory imaging and biological endpoints, descriptive summaries will be provided for all variables but inferential tests will not be performed.

##### Missing data

For response variables (including the primary outcome) patients who discontinue study medication for lack of efficacy will be considered non-responders from that point forward. In all other instances, missing data will be addressed using multiple imputation. This technique assumes the data are missing at random; additional sensitivity analyses will include complete case analysis, best case/worst case single imputation, and imputation of a range of values to test the possible implications for the study conclusions if data are missing not at random.

### Ethical approval

Ethical approval was obtained from Leeds West Research Ethics Committee (REC reference number: 10/H1307/138).

## Discussion

This pragmatic, randomised phase IV, open-label study is comparing first line ETN and MTX combination therapy with a TT regimen starting with MTX monotherapy regimen with sDMARD escalation followed by ETN and MTX at week 24 if the treatment target of remission (or LDA with physician impression of remission) is not achieved.

The fundamental objective of this study is to determine whether TNFi instituted as first-line therapy in early RA confers better outcomes (including depth of remission and exploratory parameters indicating normalisation of immune system aberration) compared to a conventional TT therapeutic approach with delayed TNFi if required. The VEDERA study will contribute to the existing evidence [37, 38] and in addition addresses the question of whether quality of response to TNFi following MTX-failure differs from that achieved with first-line use, prior to MTX exposure. The accurate imaging aligned with the clinical assessments will also ensure correct categorisation of patient response, to address the weaknesses of clinical composite measures for assessing remission [39].

Although the planned sample size (*n*=120) is relatively small, the study is adequately powered to detect a clinically meaningful difference in remission rate, based on existing published data. Secondary outcomes are expected to be correlated with the primary outcome and we therefore anticipate the effect size to be similar for these variables, which are considered supportive of the primary hypothesis. For exploratory endpoints the focus will be purely descriptive; the sample size has been inflated to help ensure that planned subgroup analyses of patients receiving delayed ETN will include sufficient patients, based on rules of thumb for pilot studies.

One of the secondary objectives of the study is remission maintenance and sustainability after ETN withdrawal. There is increasing published data regarding this [40], and while this study does not primarily seek to investigate drug tapering following remission, it will provide additional insights. Finally, the biosamples collected will allow investigation of the pathobiology of disease, mechanisms of drug action and predictors of response and remission in order to enable more effective tailoring of therapy in clinical practice.

## Abbreviations

ACPA: Anti-cyclic citrullinated peptide antibody
ACR: American college of rheumatology
CDAI: Clinical disease activity index
CV: Cardiovascular
HS-CRP: High sensitivity C-reactive protein
DAS: Disease activity score
DMARD: Disease modifying anti-rheumatic drug
sDMARD: synthetic DMARD
bDMARD: biological DMARD
DCE-MRI: Dynamic contrast enhanced magnetic resonance imaging
ESR: Erythrocyte sedimentation rate
ETN: Etanercept
EULAR: European league against rheumatism
EQ-5D: Euro quality of life five dimensions questionnaire
HAQ: Health assessment questionnaire
HCQ: Hydroxychloroquine
HRUS: High resolution ultrasonography
LDA: Low disease activity
MTX: Methotrexate
MRI: Magnetic resonance imaging
NICE: National institute of clinical excellence
NSAID: Nonsteroidal anti-inflammatory drug
OMERACT: Outcome measures in RA clinical trials
RA: Rheumatoid arthritis
RF: Rheumatoid factor
RAQoL: RA quality of life questionnaire
RA-WIS: RA work instability questionnaire
RAMRIS: RA magnetic resonance imaging score
SDAI: Simplified disease activity index
SSZ: Sulfasalazine
TNF: Tumour necrosis factor
TNFi: Tumour necrosis factor inhibitor
TT: Treat to target (current recommendations for best practice: rapid escalation of treatment to achieve a pre-defined target of disease control, with monthly disease activity assessment)
VAS: Visual analogue scale
